# Safety of Insulin Degludec/Insulin Aspart in Patients with Diabetes Mellitus over a Period of 1 Year during Routine Clinical Care in India: SMART (Study of Management of Diabetes with Ryzodeg™ Treatment)

**DOI:** 10.3390/medsci10010001

**Published:** 2021-12-21

**Authors:** Jothydev Kesavadev, Ambanna Gowda, Harish Kumar, Sadasiva Rao Yalamanchi, Sailesh Lodha, Kiran Pal Singh, Debasis Basu, Arthur Asirvatham, Navneet Shah, Muzammil Khan Pathan, Manjunatha Revanna, Jagat Jyoti Mukherjee

**Affiliations:** 1 Jothydev’s Diabetes Research Centre, Trivandrum 695032, India; 2 Fortis Hospital, Cunningham Road, Bengaluru 560052, India; dr.ambanagowda@gmail.com; 3 Amrita Institute of Medical Sciences, Kochi 682041, India; harishkumar@aims.amrita.edu; 4 Yalamanchi Hospitals & Research Centres Pvt Ltd., Vijayawada 520002, India; drsada@yahoo.com; 5 Eternal Heart Care Centre, Jaipur 302017, India; saileshlodha@gmail.com; 6 Fortis Hospital, Mohali 160062, India; drkp1292@gmail.com; 7 Apollo Gleneagles Hospital, Kolkata 700067, India; drdbasu@yahoo.com (D.B.); jjmukh@gmail.com (J.J.M.); 8 Arthur Asirvatham Hospital, Madurai 625020, India; ajasirvathamresearch@yahoo.in; 9 Sterling Hospital, Ahmedabad 380052, India; navneetnshah2000@gmail.com; 10 Novo Nordisk India Private Limited, Bengaluru 560066, India; phmk@novonordisk.com (M.K.P.); mjnr@novonordisk.com (M.R.)

**Keywords:** diabetes mellitus, glycemic control, IDegAsp, India, insulin therapy, safety

## Abstract

This post-authorization study was conducted to evaluate the safety of insulin degludec/insulin aspart (IDegAsp) in adult patients with diabetes mellitus (DM) during routine clinical care under a real-world setting in India. Eligible patients received IDegAsp for a minimum of 12 months during routine clinical management. Data were collected at 0, 3, 6, and 12 months. In total, 1029 adult patients with DM were included; 65.2% (n = 671) were men; mean age was 55.0 ± 12.2 years, and the mean duration of diabetes mellitus was 10.8 ± 7.4 years. Thirty adverse events were reported in 23 patients (2.2%) during the follow-up: two adverse events in two patients were serious with fatal outcomes, which were unrelated to IDegAsp use. At baseline, there were 176 confirmed hypoglycemic events in 67 (6.7%) patients while they were on their previous treatment options. At 12 months of treatment with IDegAsp, 11 confirmed hypoglycemic events were reported in 11 (1.1%) patients since the previous visit; there were no reported episodes of severe hypoglycemia. Mean glycosylated hemoglobin value reduced from 9.5% ± 1.8% at baseline to 7.7% ± 1.1% at 12 months. This study showed the safety of IDegAsp in patients with diabetes mellitus over a period of 1 year during routine clinical care.

## 1. Introduction

The burden of diabetes mellitus (DM) is on the rise globally; at 77 million, India ranks second for the number of people with DM in the age group of 20–79 years, and by 2045, this number is estimated to rise to 134.2 million [1].

Insulin is the mainstay of therapy against type 1 DM (T1DM) and is also necessary for people with type 2 DM (T2DM) under certain circumstances at the diagnosis of T2DM, and particularly following the inability of oral anti-diabetic drugs (OADs) to maintain glycemic control [2,3]. The joint RSSDI-ESI (Research Society for the Study of Diabetes in India—Endocrine Society of India) clinical practice recommendations 2020 suggest that insulin initiation with once-daily co-formulation/premix or basal insulin should be considered if the glycosylated hemoglobin (HbA1c) value is not on target despite three OADs [4]. Basal insulins primarily control fasting plasma glucose (FPG), and overall HbA1c values might still exceed target value owing to uncontrolled post-prandial plasma glucose (PPG) excursions [5,6]. PPG excursion is of concern among people with DM in India, owing to relatively high dietary carbohydrate intake that can lead to relatively higher contribution of PPG to hyperglycemia [7,8]. Post-prandial hyperglycemia can be corrected by using prandial insulins [9]. However, introduction of an additional injection may reduce patient compliance [10]. IDegAsp is a co-formulation of 70% insulin degludec and 30% insulin aspart, offering total control of fasting and post-prandial glucose levels [11]. Phase 2 and 3 studies have demonstrated safety and efficacy of the IDegAsp compared with basal, basal-plus, basal-bolus, and analog premix insulin regimens [11,12,13,14,15,16]. This post-marketing surveillance (PMS) study of IDegAsp (Ryzodeg™), titled “Study of MAnagement of diabetes with Ryzodeg™ Treatment (SMART)”, was conducted as part of a regulatory requirement. It aimed at assessing the safety of IDegAsp during routine clinical care under a real-world setting in India.

## 2. Materials and Methods

### 2.1. Participants

Men and women with DM aged >18 years who were scheduled to start treatment with IDegAsp based on the clinical judgement of the investigator during routine care were included in this study. Patients previously on IDegAsp therapy or those participating in another study were excluded. Patients with mental incapacity, unwillingness to participate, or language barriers precluding adequate understanding or cooperation were excluded. Women who were pregnant, were breast-feeding, or had the intention of becoming pregnant within 12 months were also excluded.

### 2.2. Study Design

This multicenter, prospective, single-arm, observational, PMS study (NCT02230618; CTRI/2015/12/006442) was conducted at 40 sites across India between November 2015 and June 2017. It was conducted as per the Declaration of Helsinki and International Conference on Harmonization-Good Clinical Practice guidelines. After obtaining approval from Institutional Review Board/Independent Ethics Committee of each participating center, the study was conducted in compliance with the protocol. All enrolled patients provided written informed consent.

At baseline (visit 1), data on demographics, medical history (DM history, prior DM treatment, history of hypoglycemia on previous treatment, and reason to start IDegAsp), concomitant medications, height, and weight were recorded. IDegAsp, marketed as Ryzodeg™ FlexTouch^®^ prefilled pen injector (100 units/mL) and available in the market by prescription, was prescribed. The decision to initiate, titrate, and intensify with IDegAsp, its dose, its timings, and frequency were based on the investigator’s discretion in line with approved Indian label. Safety data, including the hypoglycemic episodes since the last visit, were collected at 3 months ± 2 weeks (visit 2), 6 months ± 2 weeks (visit 3), and finally at 12 months ± 2 weeks (visit 4). Hypoglycemic events while on previous treatment were recorded based on patient recall of confirmed (blood glucose < 56 mg/dL) or severe hypoglycemic (requiring third-party assistance) events in the immediate 4-week period before starting IDegAsp. Because this was a non-interventional study, meticulously structured self-monitoring of blood glucose was not mandatory, and hypoglycemic events during the 12-month treatment with IDegAsp were recorded based on patient recall of confirmed or severe hypoglycemic events during follow-up visits.

### 2.3. Safety Assessments

During the 1-year study period, patients reported safety incidences were evaluated and categorized by physicians as follows: adverse events (AEs), serious adverse events (SAEs), adverse drug reactions (ADRs), serious ADRs, and confirmed or severe hypoglycemia. Additionally, causality (probable, possible, or unlikely), severity (mild, moderate, or severe), and outcome (recovered/resolved, recovering/resolving, recovered/resolved with sequelae, not recovered/not resolved, fatal, or unknown) of AEs/ADRs were recorded.

### 2.4. Assessment of the Change in HbA1c, FPG, and PPG

Endpoints included mean change in HbA1c, FPG, and PPG values from baseline at 3, 6, and 12 months in the overall population. The additional analysis included mean change in HbA1c, FPG, and PPG values from baseline to 3, 6, and 12 months, stratified by previous treatment (OADs or insulin). Given the non-interventional nature of this study, the most recent fasting and post-prandial glucose value was captured for assessment at different time points which could either be a lab or glucometer-based measurement.

### 2.5. Statistical Analyses

Assuming a 20% dropout rate, a sample size of 1000 patients was determined to provide a power of 80% to detect at least one AE that occurs with an incidence of 2 in 1000 patients, or approximately 6 events with an incidence of 1 in 100 patients. The descriptive statistics for continuous variables were presented with the number (n) of observations, the number of missing observations, mean, standard deviation (SD), median, and minimum and maximum of the range. For categorical data, descriptive statistics were presented using counts and percentages. All the patients who received at least one dose of IDegAsp during the study were included in the safety analysis set (SAS), and patients who had at least one post-baseline measurement available for HbA1c, FPG, or confirmed hypoglycemic event were included in the efficacy analysis set (EAS). A paired two-sided t-test at a 5% significance level was used to evaluate the changes in HbA1c, FPG, PPG, and confirmed hypoglycemic events by the visit. Statistical analyses were performed using Statistical Analysis Software (Version 9.4).

## 3. Results

### 3.1. Patient Disposition and Baseline Characteristics

All 1029 patients with DM were included in the SAS, and 1003 were included in the EAS, of whom 971 (94.4%) completed the study (Table 1 and Figure S1). 

The mean (±SD) age of the study participants was 55.0 ± 12.2 years, and majority of them were male (n = 671, 65.2%). The mean duration of DM was 10.8 ± 7.4 years. Based on EAS, before visit 1, 730 patients (72.8%) were on OAD(s) and 273 (27.2%) were on insulin therapy ± OAD(s). Along with other concomitant drugs, OAD(s) in use at baseline included metformin (n = 766, 74.4%), sulphonylureas (n = 599, 58.2%), alpha-glucosidase inhibitors (n = 203, 19.7%), meglitinides (n = 2, 0.2%), thiazolidinediones (n = 86, 8.4%), dipeptidyl peptidase-4 (DPP-4) inhibitors (n = 363, 35.3%), and sodium-glucose co-transporter-2 (SGLT-2) inhibitors (n = 16, 1.6%) apart from glucagon-like peptide-1 receptor agonists (GLP-1 RAs) (n = 5, 0.5%). Treatment with IDegAsp and its continuation over a period of 12 months resulted in a change in use of OAD(s) such that at the last visit, OAD(s) in use were metformin (n = 684, 66.5%), sulphonylureas (n = 456, 44.3%), alpha-glucosidase inhibitors (n = 159, 15.5%), meglitinides (n = 6, 0.6%), thiazolidinediones (n = 58, 5.6%), DDP-4 inhibitors (n = 283, 27.5%), and SGLT-2 inhibitors (n = 50, 4.9%) apart from GLP-1 RAs (n = 5, 0.5%). Other concomitant medications in use reported in ≥5% of patients are mentioned in Table S1. In terms of insulin therapy, at baseline, 124 patients (12.1%) were on basal insulins, 135 (13.1%) on premix insulins, and 95 (9.2%) on bolus insulins with or without basal/premix insulins before being enrolled in this study. Subsequently, 158 patients (15.4%) at visit 2 (at 3 months), 160 patients (15.5%) at visit 3 (at 6 months), and 162 patients (15.7%) at visit 4 (at 12 months) were on bolus insulin along with IDegAsp. 

Microvascular and macrovascular complications were recorded at baseline as shown in Table 2. The most common reason cited by treating physicians for starting IDegAsp was to improve HbA1c (n = 895, 87.0%) (Table 2).

### 3.2. Safety and Tolerability

#### 3.2.1. Adverse Events

Of the 1029 patients, 23 (2.2%) patients had 30 AEs and 5 (0.5%) patients had >1 AEs. AEs reported in ≥2 patients were pyrexia (n = 5), fatigue (n = 2), upper respiratory tract infection (n = 2), dizziness (n = 2), and muscle spasm (n = 2) (Table 3). On the severity scale, 19 (1.8%) patients had 25 mild AEs, 1 had 1 moderate AE, and 3 had 4 severe AEs (cardiogenic shock, death, hyperglycemia, and accelerated hypertension). Most of the events (19 AEs) in 16 patients were resolved, 8 AEs in 5 patients were unresolved, 2 were fatal, and 1 had an unknown outcome. Of the total AEs, the majority (23 AEs out of 30 AEs, in 20 patients) were unlikely to be related to the study drug; 5 AEs in 3 patients were probably and 2 AEs in 2 patients were possibly related to the study drug. IDegAsp dose was reduced in 2 patients (hypoglycemia and dizziness); it was withdrawn in 2 patients (weight gain, hyperglycemia, and accelerated hypertension). A total of 2 patients had SAEs leading to death; however, both were unrelated to the study drug (Table 3).

#### 3.2.2. Adverse Drug Reactions 

A total of 7 ADRs were reported in 5 (0.5%) patients during the study: fatigue, dizziness, weight gain, hyperglycemia, increased appetite, and injury. A total of 4 (0.4%) patients had 1 ADR each, and 1 (0.1%) patient had 3 ADRs. On the severity scale, ADRs were mild in 3 patients (5 ADRs) and moderate and severe in 1 patient each. A total of 3 ADRs observed in 3 patients were resolved, three ADRs in 1 patient did not resolve, and the outcome of 1 ADR in 1 patient was unknown. The study drug was withdrawn in 2 patients, the dose was reduced in 1, and the dose remained unchanged in 1 patient. No serious ADRs were reported in this study (Table 3).

#### 3.2.3. Hypoglycemic Events

Overall, 24 severe hypoglycemic events were experienced by 17 patients (1.7%) before visit 1 (baseline). No episodes of severe hypoglycemia were reported at any follow-up visit during the 12-month study period. In total, 176 confirmed hypoglycemic events (blood glucose <56 mg/dL) were reported in 67 patients (6.7%) before visit 1 (baseline). There were 11 confirmed hypoglycemic events in 11 patients (1.09%) since the previous visit 3, as reported at the last visit (Table 4).

#### 3.2.4. Clinical Laboratory Findings and Vital Parameters

During all visits, measured laboratory parameters, including total cholesterol and triglycerides were within the normal range, and the differences were not significant (Table S2). The average mean weight was 73.5 ± 12.5 kg at 12 months compared with 73.2 ± 12.5 kg at baseline. The mean total daily dose of IDegAsp used at baseline was 20.4 ± 10.9 units, which increased to 22.2 ± 27.9 units by the last visit at 12 months.

### 3.3. Change in HbA1c, FPG, and PPG

#### 3.3.1. Glycosylated Hemoglobin 

**Figure 1:**
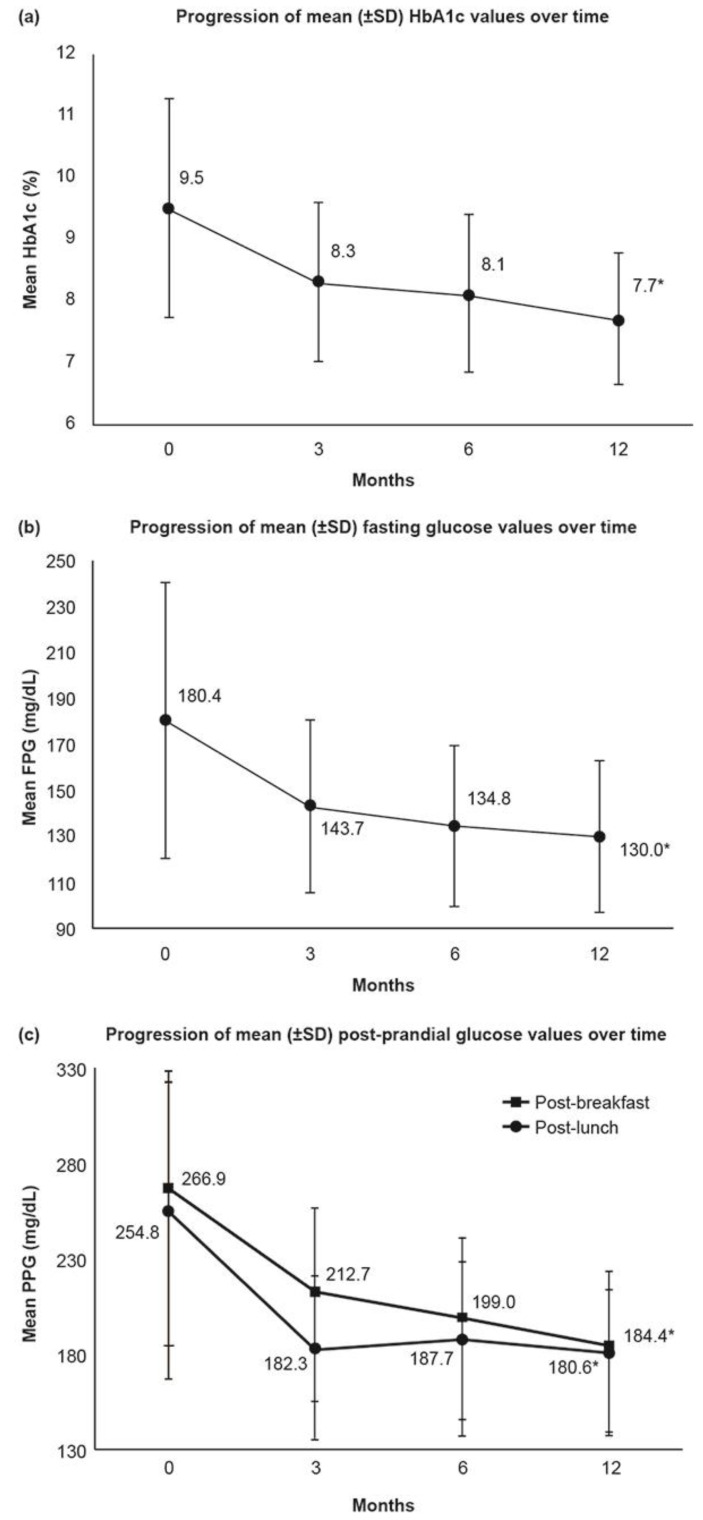
(**a**) Progression of mean (±SD) HbA1c values over time; (**b**) Progression of mean (±SD) fasting glucose values over time; (**c**) Progression of mean (±SD) post-prandial glucose values over time. FPG: Fasting plasma glucose; HbA1c: Glycosylated hemoglobin; PPG: Post-prandial plasma glucose; SD: Standard deviation. Note: All values are presented as mean ± SD. * (*p* < 0.0001).

The HbA1c values (mean ± SD) decreased from 9.5% ± 1.8% at baseline to 7.7% ± 1.1% at 12 months (Figure 1a) (Table S3). Within 3 months of treatment, the HbA1c value reduced by 1.0% ± 1.2%, and a maximum reduction of 1.7% ± 1.6% was observed at 12 months. A similar trend was observed when mean reductions in HbA1c were stratified by earlier medication use. The HbA1c value (mean ± SD) in the OAD-treated patients decreased from 9.3% ± 1.7% at baseline to 7.6% ± 1.0% at 12 months. Similarly, the HbA1c value (mean ± SD) decreased from 9.9% ± 1.9% at baseline to 8.1% ± 1.1% at 12 months in patients who were previously on insulin. The reductions in HbA1c values at each visit were statistically significant (*p* < 0.0001) when compared with the HbA1c value at baseline in overall and both OAD- and insulin-treated groups (Table S3).

#### 3.3.2. Fasting Plasma Glucose/Fasting Blood Glucose

The FPG value (mean ± SD) decreased from 180.4 ± 59.7 mg/dL to 130.0 ± 33.1 mg/dL at 12 months (Figure 1b) (Table S3). The decrease in FPG values at each visit was statistically significant compared with the baseline value in overall and both OAD and insulin-treated groups (*p* < 0.0001).

#### 3.3.3. Post-Prandial Plasma Glucose

The post-breakfast PPG value (mean ± SD) reduced from 266.9 ± 77.8 mg/dL at baseline to 184.4 ± 47.2 mg/dL at 12 months. Similarly, the post-lunch PPG value (mean ± SD) reduced from 254.8 ± 84.0 mg/dL at baseline to 180.6 ± 40.1 mg/dL at 12 months (Figure 1c). The reductions in PPG values post-breakfast and post-lunch at all time points were statistically significant (*p* < 0.0001) compared with the baseline value. Very few patients had their post-dinner values recorded, and the significance of the change in post-dinner PPG values could not be determined (Table S3). Similar trends in decline of PPG values (*p* < 0.0001) were observed in patients receiving OADs and insulin previously.

## 4. Discussion

IDegAsp (Ryzodeg™) received its first regulatory approval by Japanese Ministry of Health in December 2012 [17]. United States Food and Drug Administration approved its use on 25 September 2015 [18]. It is available in the Indian market since January 2015 [19]. IDegAsp provides total (FPG + PPG) glycemic control with simplicity and convenience [11,16,20]. The basal insulin component in IDegAsp, i.e., insulin degludec has a long half-life and achieves a steady state within 2–3 days [21,22]. The efficacy and safety of IDegAsp had already been established based on the data obtained from earlier phase 2 and 3 studies [11,12,13,14,15]. However, to ensure its safety in the Indian population, Central Drugs Standard Control Organization (CDSCO) recommended a local post-marketing safety study. Hence, this multicenter, prospective, single-arm, observational study was conducted in patients who were scheduled to start treatment with IDegAsp as a part of standard routine clinical care. A one-year study duration was considered sufficient to determine any ADR associated with IDegAsp use.

In this study, patients with DM receiving IDegAsp as a part of routine clinical care for 12 months had acceptable tolerability and showed a significant and consistent decrease in fasting and PPG values along with a reduction in HbA1c value by 1.7% ± 1.6% at 12 months. Similar HbA1c reductions in the OAD (9.3% to 7.6%) and the insulin (9.9% to 8.1%) treated groups were seen in this study which could possibly be due to a higher baseline HbA1c in the insulin treated group. Timely dose optimization and insulin intensification, which is usually missed, could have resulted in a more impressive glycemic control. As per the label, treating physician could use IDegAsp once daily or twice daily which can explain the reason behind improvement in more than one post-meal hyperglycemia. Some part of PPG reduction across different meals can also be due to improvement in pre-meal glucose values. Prominent reasons cited by physicians for starting IDegAsp included better glycemic control, lesser risk of hypoglycemia, and flexibility in the timing of dosing. 

The AEs observed in this study were few, mostly mild to moderate in nature, recoverable, and mostly unrelated to the study drug. Most observed AEs were pyrexia, fatigue, upper respiratory tract infection, dizziness, and muscle spasm. Two SAEs reported in this study were fatal; however, they were unrelated to the study drug. Confirmed hypoglycemic events were few, and there was no instance of the severe hypoglycemic events throughout the study. In a real-world study conducted in 152 Indian patients with T2DM, only five non-severe hypoglycemic events were reported whilst receiving IDegAsp for a mean duration of 10.3 months [23]. Unlike conventional premix insulins, the risk of hypoglycemia has been shown to be significantly lower with IDegAsp. This may be due the absence of shoulder effect with IDegAsp and a flat and more predictable glucose lowering effect of insulin degludec component [24]. In a 26-week, phase 3, open-label trial conducted in Japanese patients with T2DM, the number of overall confirmed hypoglycemic events (134 vs. 190) and nocturnal confirmed hypoglycemic events (27 vs. 37) were numerically lower in the once-daily IDegAsp group than in the once-daily insulin glargine U100 group. In both the treatment groups, nasopharyngitis and diabetic retinopathy were the most frequently reported AEs [11]. 

In this study group, insulin therapy with IDegAsp significantly reduced HbA1c by 1% ± 1.2% at 3 months and 1.7% ± 1.6% at 12 months. Similarly, the FPG values significantly reduced after 12 months, irrespective of the baseline anti-diabetic therapy with OAD(s) or insulin. The decrease in PPG after breakfast and lunch was also consistent and statistically significant at 12 months. Our results are in line with previous Indian real-world study that reported HbA1c reduction (mean ± SD) from 9.5% ± 1.3% at baseline to 7.5% ± 0.4%, which is comparable to a reduction from a baseline HbA1c value of 9.5% ± 1.8% to 7.7% ± 1.1% in this study [25]. In global clinical studies, the decrease in the mean HbA1c value varied from 1.1% to 1.7% over 26 weeks compared with a mean decrease of 1.7% over 52 weeks in this study [11,15,16,24]. The decrease in FPG reported in an Indian observational study (154.1 ± 33.3 mg/dL at baseline to 102.2 ± 12.8 mg/dL at 12 months) is comparable to that noted in our study (180.4 ± 59.7 mg/dL at baseline to 130.0 ± 33.1 mg/dL at 12 months) [25].

The strength of this study is the long duration of follow-up of 1 year involving a large number of patients from routine clinical practice with a low dropout rate. However, expectedly, there are a few limitations in this real-world PMS study. Although it was emphasized in the protocol to determine and report all confirmed and severe hypoglycemic events, a few hypoglycemic events could be missed because unlike in most randomized clinical trial, the patients in the present study were not mandated to regularly self-monitor blood glucose levels and the hypoglycemic events were reported based on patient recall. Therefore, hypoglycemic events could be under-reported. Moreover, in the absence of a comparator arm, the reduction in mean HbA1c value from 9.5% ± 1.8% to 7.7% ± 1.1% and the hypoglycemic events as seen in this study reflect the combined effect of IDegAsp and other concomitant anti-diabetic medications in use. Lastly, the type of diabetes and daily frequency of IDegAsp administration were not documented for all patients.

Indian patients receiving IDegAsp for managing DM showed acceptable tolerability with no new safety signals. Most of the safety events were mild to moderate and resolvable and did not result in study drug discontinuation. Improved glycemic control (HbA1c, FPG, and PPG) without compromise on the safety was noted in patients on IDegAsp. Most of the patients were prescribed IDegAsp to improve glycemic control, to reduce the risk of hypoglycemia, and owing to the need for flexibility in the timing of injection. 

## 5. Conclusions

This prospective, non-interventional study of IDegAsp confirms its long-term safety and tolerability with good improvements in glycemic control when used under routine clinical practice conditions.

## Figures and Tables

**Table 1:** Summary of patient disposition.

Category	Enrolled Population (N = 1029) n (%)
Patients in safety analysis set ^a^	1029 (100)
Patients in efficacy analysis set ^b^	1003 (97.5)
Patients completed study	971 (94.4)
Patients discontinued study	58 (5.6)
Reason for discontinuation ^c^	
Lost to follow-up	41 (4.0)
Adverse drug reaction	0
IDegAsp discontinued	12 (1.2)
Other	6 (0.6)

IDegAsp: Insulin degludec/insulin aspart; N: Total number of patients; n: number of patients in a specified category. ^a^ Those who received at least one dose of IDegAsp during the study ^b^ Those who had at least one post-baseline measurement available for glycosylated hemoglobin, fasting plasma glucose, or confirmed hypoglycemic event ^c^ One patient discontinued for more than one reason.

**Table 2:** Demographics and other baseline medical history.

Parameters	N = 1029
Men, n (%)	671 (65.2)
Age (completed years), mean ± SD	55.0 ± 12.2 ^a^
Hip circumference (cm), mean ± SD	98.7 ± 12.5 ^b^
Waist circumference (cm), mean ± SD	95.2 ± 11.6 ^c^
Weight (kg), mean ± SD	73.2 ± 12.5 ^d^
Microvascular complications, n (%)	
Peripheral neuropathy	214 (20.8)
Nephropathy	74 (7.2)
Autonomic neuropathy	72 (7.0)
Retinopathy	63 (6.1)
Macrovascular complications, n (%)	
Coronary heart disease	76 (7.4)
Stroke	22 (2.1)
Macroangiopathy including peripheral vascular disease	20 (1.9)
Reasons to start IDegAsp, n (%)	
Improve HbA1c	895 (87.0)
Improve PPG	645 (62.7)
Improve FPG	593 (57.6)
Reduce risk of hypoglycemia	413 (40.1)
Need for flexibility in timing of injection	228 (22.2)
Patients dissatisfaction with previous therapy	153 (14.9)
Side effects from previous therapy	27 (2.6)
Improve weight control	126 (12.2)
Improve beta cell function	73 (7.1)
Other	6 (0.6)

FPG: Fasting plasma glucose; HbA1c: Glycosylated hemoglobin; IDegAsp: Insulin degludec/insulin aspart; N: Number of total patients; n: Number of patients in a specified criterion; %: n/N; PPG: Post-prandial glucose; SD: Standard deviation. ^a^ n = 1027; ^b^ n = 474; ^c^ n = 682; ^d^ n = 1028. Note: Patients may have more than one reason for starting IDegAsp therapy.

**Table 3:** Summary of adverse events and adverse drug reaction (safety analysis set).

	Adverse Event (N = 1029) n (%)	Adverse Drug Reaction (N = 1029) n (%)
Total number of AEs/ADRs reported	30	7
Patients reporting any AEs/ADRs	23 (2.2) {30}	5 (0.5) {7}
Patients reporting 1 AEs/ADRs	18 (1.7) {18}	4 (0.4) {4}
Patients reporting >1 AEs/ADRs	5 (0.5) {12}	1 (0.1) {3}
Serious AEs/ADRs	2 (0.2) {2}	-
Life-threatening AEs/ADRs	2 (0.2) {2}	-
Severity		
Mild	19 (1.8) {25}	3 (0.3) {5}
Moderate	1 (0.1) {1}	1 (0.1) {1}
Severe	3 (0.3) {4}	1 (0.1) {1}
Outcome of AEs		
Recovered/Resolved	16 (1.6) {19}	3 (0.3) {3}
Not recovered/Not resolved	5 (0.5) {8}	1 (0.1) {3}
Recovering/Resolving	-	-
Fatal	2 (0.2) {2}	-
Recovered/Resolved with sequelae	-	-
Unknown	1 (0.1) {1}	1 (0.1) {1}
Causality		
Probable	3 (0.3) {5}	-
Possible	2 (0.2) {2}	-
Unlikely	20 (1.9) {23}	-
Actions taken to study product(s) due to adverse event/adverse drug reaction		
Drug interrupted	-	-
Drug withdrawn	2 (0.2) {3}	2 (0.2) {2}
Dose reduced	2 (0.2) {2}	1 (0.1) {1}
Dose increased	-	-
Dose not changed	7 (0.7) {12}	1 (0.1) {3}
Unknown	-	-
Not applicable	12 (1.2) {13}	1 (0.1) {1}
AEs reported in ≥2 patients or number of ADRs reported		
Fatigue	2 (0.2) {2}	1 (1.0) {1}
Pyrexia	5 (0.5) {5}	-
Upper respiratory tract infection	2 (0.2) {2}	-
Dizziness	2 (0.2) {2}	1 (0.1) {1}
Muscle spasms	2 (0.2) {2}	-
Weight gain	-	1 (0.1) {1}
Hyperglycemia	-	1 (0.1) {1}
Increased appetite	-	1 (0.1) {2}
Injury, poisoning, and procedural complications	-	1 (1.0) {1}

ADR: Adverse drug reaction; AE: Adverse event; N: Total number of patients; n: Total number of patients in a specified criterion. Note: Numbers in {} indicate the number of AEs/ADRs. Patient may have reported more than one AE/ADR.

**Table 4:** Episodes of confirmed and severe hypoglycemia.

Parameters	Visit 1 (Baseline)	Visit 2 (3 Months ±2 Weeks)	Visit 3 (6 Months ±2 Weeks)	Visit 4 (12 Months ±2 Weeks)
Confirmed hypoglycemia	67 (6.7%) {176}	12 (1.2%) {28}	15 (1.5%) {17}	11 (1.1%) {11}
Severe hypoglycemia	17 (1.7%) {24}	Nil	Nil	Nil

n (%) [Number of total episodes]; n: Number of patients who reported experiencing hypoglycemia since last visit; % = Proportion of patients. Note: Numbers in {} indicate the actual number of episodes experienced by the patients.

## Data Availability

Request can be raised for de-identified data through the corresponding author.

## Supplementary Materials

The following supporting information can be downloaded at: https://www.mdpi.com/article/10.3390/medsci10010001/s1, Figure S1: Patient disposition; Table S1: Other concomitant medications in use reported in ≥5% of patients (safety analysis set); Table S2: Summary of laboratory parameters (safety analysis set); Table S3. Summary of HbA1c, FPG, and PPG values over study duration (efficacy analysis set).
