# Supplementary material for: Safety of Insulin Degludec/Insulin Aspart in Patients with Diabetes Mellitus over a Period of 1 Year during Routine Clinical Care in India: SMART (Study of Management of Diabetes with Ryzodeg™ Treatment)
Source: Med Sci (Basel). 2021 Dec 21;10(1):1. doi: 10.3390/medsci10010001 (PMC8788540; doi:10.3390/medsci10010001)
Supplement: Supplementary file 1 [file medsci-10-00001-s001.zip › medsci-1312630-supplementary.pdf]

medical sciences

MDPI

Supporting Information

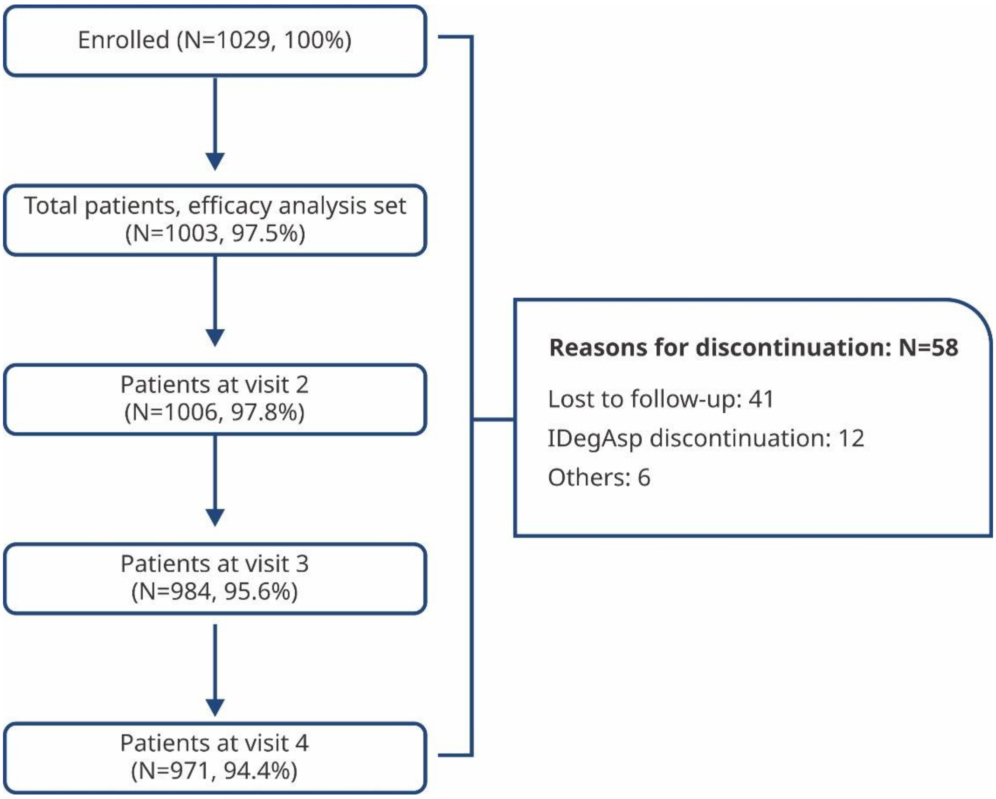

Figure S1. Patient disposition. N: Number of patients; IDegAsp: Insulin degludec/Insulin aspart; One patient discontinued for more than one reason

Table S1. Other concomitant medications in use reported in ≥5% of patients (safety safety analysis set)

**Concomitant medications**	**(N = 1029) n (%)**
Rosuvastatin	238 (23.1%)
Atorvastatin	228 (22.2%)
Telmisartan	209 (20.3%)
Cholecalciferol	124 (12.1%)
Levothyroxine	115 (11.2%)
Acetylsalicylic acid	91 (8.8%)
Metoprolol	65 (6.3%)
Amlodipine	56 (5.4%)
Ramipril	51 (5.0%)

N: Number of total patients, n: Number of patients in a specified criterion

Table S2. Summary of laboratory parameters (safety analysis set)

Parameters	Visit 1 (Baseline)	Visit 2 (3 months ± 2 weeks)	Visit 3 (6 months ± 2 weeks)	Visit 4 (12 months ± 2 weeks)
Total cholesterol (mg/dL)	173.1 ± 45.9 (498)	160.9 ± 36.7 (259)	156.0 ± 35.0 (305)	148.0 ± 35.4 (364)
HDL-cholesterol (mg/dL)	44.6 ± 12.2 (480)	46.2 ± 11.1 (249)	45.6 ± 12.3 (282)	47.2 ± 14.1 (354)
LDL-cholesterol (mg/dL)	97.9 ± 37.7 (478)	88.9 ± 31.0 (249)	82.5 ± 31.3 (283)	77.7 ± 29.8 (353)
Triglycerides (mg/dL)	164.2 ± 103.8 (494)	127.2 ± 46.6 (252)	128.9 ± 48.9 (300)	119.0 ± 51.0 (352)

HDL: High-density lipoprotein; LDL: Low-density lipoprotein; n: number of patients in a specified criterion; SD: Standard deviation

Note: Data are presented as mean ± SD (n).

Table S3. Summary of HbA1c, FPG, and PPG values over study duration (efficacy analysis set).

**Parameter**		**Visit 1 (Baseline)**	**Visit 2 (3 months ± 2 weeks)**	**Visit 3 (6 months ± 2 weeks)**	**Visit 4 (12 months ± 2 weeks)**
HbA1c value		9.5 ± 1.8 (903)	8.3 ± 1.3 (629)	8.1 ± 1.3 (705)	7.7 ± 1.1 (892)
HbA1c by previous medication (%)	OAD	9.3 ± 1.7 (678)	8.2 ± 1.3 (498)	7.9 ± 1.2 (534)	7.6 ± 1.0 (650)
	Insulin	9.9 ± 1.9 (225)	8.7 ± 1.2 (131)	8.7 ± 1.4 (171)	8.1 ± 1.1 (242)
FPG (mg/dL)		180.4 ± 59.7 (792)	143.7 ± 37.8 (748)	134.8 ± 35.2 (768)	130.0 ± 33.1 (896)
FPG by previous medication (mg/dL)	OAD	181.1 ± 56.7 (587)	144.7 ± 37.9 (560)	133.4 ± 32.1 (579)	128.6 ± 32.4 (659)
	Insulin	178.3 ± 67.6 (205)	140.7 ± 37.5 (188)	139.2 ± 43.0 (189)	133.9 ± 34.6 (237)
PPG (mg/dL)	Post-breakfast	266.9 ± 77.8 (476)	212.7 ± 54.8 (461)	199.0 ± 51.7 (468)	184.4 ± 47.2 (515)
	Post-lunch	254.8 ± 84.0 (175)	182.3 ± 46.6 (161)	187.7 ± 49.2 (175)	180.6 ± 40.1 (253)
	Post-dinner	216.3 ± 58.0 (3)	160.0 ± 28.6 (17)	192.3 ± 32.6 (12)	164.9 ± 36.2 (10)
PPG by previous medication	Post-breakfast	277.7 ± 73.6 (309)	211.6 ± 56.6 (298)	196.8 ± 48.7 (301)	183.8 ± 47.5 (348)
	Post-lunch	257.6 ± 86.6 (147)	187.8 ± 47.1 (122)	188.5 ± 52.1 (138)	179.6 ± 38.5 (178)

(OAD) (mg/dL)	Post-dinner	200.0 (1)	169.5 ± 17.0 (4)	170.7 ± 2.3 (3)	141.8 ± 4.2 (4)
PPG by previous medication (insulin) (mg/dL)	Post-breakfast	247.0 ± 81.5 (167)	214.6 ± 51.4 (163)	203.0 ± 56.8 (167)	185.6 ± 46.7 (167)
	Post-lunch	240.1 ± 68.4 (28)	165.1 ± 40.7 (39)	184.9 ± 37.0 (37)	182.7 ± 43.9 (75)
	Post-dinner	224.5 ± 79.6 (2)	157.1 ± 31.2 (13)	199.4 ± 35.0 (9)	180.3 ± 40.4 (6)

FPG: Fasting plasma glucose; HbA1c: Glycosylated hemoglobin; n: Number of patients in a specified criterion; OAD: Oral antidiabetic drug, PPG: Post-prandial plasma glucose; SD: Standard deviation

Note: Data are presented as mean ± SD (n).
